# Cognitive behavioral game design: a unified model for designing serious games

**DOI:** 10.3389/fpsyg.2014.00028

**Published:** 2014-02-03

**Authors:** Katryna Starks

**Affiliations:** University of the Sunshine CoastSippy Downs, QLD, Australia

**Keywords:** social cognitive theory, multiple intelligences, video games, game design, behavior change, goal setting, serious games, educational games

## Abstract

Video games have a unique ability to engage, challenge, and motivate, which has led teachers, psychology specialists, political activists and health educators to find ways of using them to help people learn, grow and change. Serious games, as they are called, are defined as games that have a primary purpose other than entertainment. However, it is challenging to create games that both educate and entertain. While game designers have embraced some psychological concepts such as flow and mastery, understanding how these concepts work together within established psychological theory would assist them in creating effective serious games. Similarly, game design professionals have understood the propensity of video games to teach while lamenting that educators do not understand how to incorporate educational principles into game play in a way that preserves the entertainment. Bandura (2006) social cognitive theory (SCT) has been used successfully to create video games that create positive behavior outcomes, and teachers have successfully used Gardner’s (1983) theory of multiple intelligences (MIs) to create engaging, immersive learning experiences. Cognitive behavioral game design is a new framework that incorporates SCT and MI with game design principles to create a game design blueprint for serious games.

## INTRODUCTION

Video games have a unique ability to engage, challenge, and motivate (Gee, 2003), which has lead teachers, psychology specialists, political activists, and health educators to find ways of using them to help people learn, grow and change. Serious games, as they are called, are defined as games that have a primary purpose other than entertainment. However, it is challenging to create games that both educate and entertain (Van Eck, 2006; Prensky, 2001; Schrand, 2008). In the health research sector, Bandura’s (2006) social cognitive theory (SCT) has been used successfully to create video games that create positive behavior outcomes, from increased fruit and vegetable intake to decreased calorie consumption; and better self-care for asthma (Baranowski et al., 2008). While game designers have embraced some psychological concepts such as flow (Csikszentmihalyi and LeFevre, 1989) and mastery (Gee, 2003), understanding how these concepts work together within established psychological theory would assist them in creating effective serious games.

Similarly, game design professionals have understood the propensity of video games to teach while lamenting that educators do not understand how to incorporate educational principles into game play in a way that preserves the entertainment (Ciavarro, 2006) or that students of today have differing educational requirements due to the prevalence of digital technology and that the current school system is no longer equipped to serve them (Prensky, 2001). However, teachers have successfully used Gardner’s (1983) theory of multiple intelligences (MIs) to create engaging, immersive learning experiences which increased student interest, performance and retention (Greenhawk, 1997; Hopper and Hurry, 2000; Schrand, 2008). Given the proper framework, educators can create the types of learning activities that both teach and entertain. They can then work with game designers to create digital, interactive versions of these activities as a basis for video games.

**FIGURE 1:**
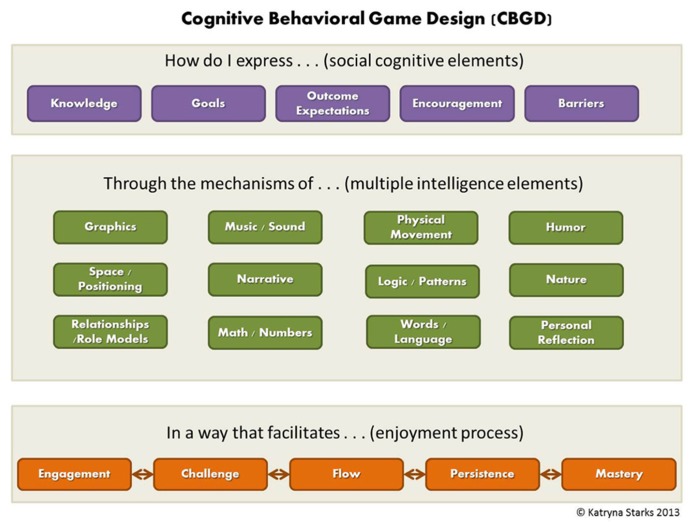
**CBGD Model**.

Cognitive behavioral game design (CBGD) is a new framework that incorporates SCT, the theory of MIs, and game design elements into a unified model that guides designers through a process to create games for learning and behavioral change. The basis of the process is the question: How do I express one or more social cognitive elements through the mechanism of one or more intelligences in a way that facilitates the enjoyment process? (**Figure 1**).

### THE SOCIAL COGNITIVE ELEMENTS

The social cognitive elements in CGBD are incorporated from Bandura’s (2004, 2006) SCT of health promotion, which outlines five elements necessary for health-related behavior change: Knowledge, self-efficacy, goals, outcome expectations, facilitators, and impediments. Following is a brief explanation of the factors, how they are expressed in SCT, and how they are expressed in CBGD.

#### Knowledge

In the SCT of health behavior, this refers to the knowledge of health risks (Bandura, 2004, 2006). However, in CBGD, it refers to knowledge the game is attempting to convey. Knowledge usually represents the overall purpose of the game, i.e., to convey the hardships faced by families in the third world, to teach children how to manage chronic disease symptoms, or to help citizens understand how their political process works. However, knowledge is not always the primary purpose. For instance, exergames often have the primary purpose of encouraging fitness using the mechanism of physical movement rather than providing knowledge about health.

#### Goals

Goals are twofold in CBGD. Goals can refer to the actual game goals (i.e., solve this puzzle, solve a mystery, take down a fictional corporation, etc.) and also the real-life outcome goals of the game (i.e., raise money for a charity, register to vote, learn a mathematical concept, understand the lifestyle of a different culture, etc.). One of the criticisms of the edutainment genre was that goals for play and goals for learning did not align (Gee, 2003; Ciavarro, 2006). When the goals for game play and the goals for learning are different, learning is removed from context (Barnes et al., 2007). Although most CBGD games are serious games, which have a goal beyond entertainment, that goal may or may not be activist. For instance, the game Third World Farmer () has a game goal of awareness. The game simulates the life and hardships of a family in the third world via obstacles, such as sickness, educational costs, and unpredictable weather. A similar game, Half the Sky (), aims to promote awareness of the hardships of women, however there is also a mechanism to donate to a women’s charity as you play the game. This is an activist goal that is designed to go beyond awareness and prompt the player to support the game’s principles outside of the game.

Another application of CBGD based goal setting within games is to create a more realistic sense of goal achievement. Visual elements can simulate the passage of time. Bar meters are common ways of depicting the passage of time in-games, but they are primarily used to show how much time a person has left before they get a losing score on the game level. In goal setting, the bar meter can show how much time has to pass before the player can perform the action again. The bar meter can also be used to show the duration of the activity itself. This mechanism is used in The Sims, where the bar meter shows longer durations for Sim activities such as exercise and learning. This simulates that the longer you have exercised or the more you have learned, the longer it takes to get to the next goal. This scaffolding of goal attainment and time to achieve mimics life.

#### Outcome expectations

Outcome expectations, like goals, have a dual expression within CBGD. One set of outcome expectations is for within game actions. For instance, if a player solves a puzzle, an outcome expectation may be that a new game area is unlocked. In a simulation style game, an outcome expectation may operate as a mimic of a real-life outcome. For instance, in the game The Sims (), game characters are fired if they miss too many days of work. Although the game designers did not state that The Sims is a teaching game about life, the element of getting fired for not working is a game outcome that parallels real-life outcome expectations for similar behavior.

#### Encouragement

In SCT, encouragement is called facilitation and/or social support. It refers to the environmental and social factors that can assist in goal attainment. An environmental facilitator would be bicycle lanes to encourage human-powered transportation, while a social factor would be an encouraging friend. Within the context of CBGD, facilitation is called encouragement, and is usually carried out within game by sounds, praise (i.e., hearing “good job!” when a mission or puzzle is completed), or even non-player characters (NPCs) acting as coaches and tutors during learning phases and giving approval for success.

#### Barriers

In SCT, the barrier element is sometimes referred to as impediments. These are factors that hinder goal attainment. Like facilitators, they can be environmental and social. In-games, barriers are often virtual manifestations of physical barriers, such as fences and rocks to leap over, or the implementation of puzzles which must be solved before the player can move to the next level. They can also be used as in-game assessments if the puzzles require that the player demonstrates knowledge that should have been learned earlier in the game. In simulation games, barriers, like outcome expectations, can be designed to mimic real-life situations. Simulated real-life barriers may help the player think about ways to overcome them in real-life.

To the extent that realistic barriers can be simulated within games, they can force the player to think through how he or she would handle the barrier in life. Barriers and challenges can also be used to show a barrier that is usually unseen. Re-mision, a game about cancer, simulates the inside of the body as the player fights cancer cells. This type of challenge in gaming can help players relate to the challenges they face in life by helping them to see them. This can be especially helpful with games that simulate health challenges that are not immediately felt, such as diabetes, which often causes few external symptoms. While barriers are an important part of behavior change, game-based barriers need to be designed in a way that develops the player’s coping skills rather than discouraging game play.

### SELF-EFFICACY – THE HIDDEN FACTOR

Self-efficacy has been shown to have a measurable effect on behavioral outcomes that is stronger than other individual social cognitive elements (Bandura, 2004, 2006), however, it is also comprised of and affected by several other elements. Self-efficacy is the belief about one’s ability to accomplish goals, and it has a symbiotic relationship with goals, social support, models, mastery, and barriers. For instance, levels of self-efficacy often define the goals that people make for themselves. When self-efficacy is high, people make more challenging goals, and vice versa (Bandura, 2004). Conversely, when several goals are attained within the same cognitive or behavioral domain, the result is a sense of mastery, which increases self-efficacy. Self-efficacy is also increased through observational learning. Observational learning is an intrinsic process in which a person learns via observation of the actions and outcomes of others or by example. This is primarily a social mechanism that occurs with role models. Role models can be people in relationship with the learner, such as parents, teachers, siblings, or peers. According to the SCT of mass communication (Bandura, 2001), media figures can also be role models. In CBGD, relationships and role models have been incorporated into the mechanisms section because the game can either facilitate real-life relationships if it is played with or discussed with others, or can provide direct models via NPCs in-game. Coping skills, or the increased ability to overcome barriers, have been incorporated into the enjoyment process as persistence. In CBGD, persistence is an internally motivated outcome of engagement, challenge and flow. Players are persistent because they want to complete the game. Mastery, described above as the completion of several goals within the same cognitive or behavioral domain, is often accompanied by a feeling of accomplishment, and also increases self-efficacy. As such, self-efficacy has not been incorporated into the model as a separate term, however, all of the elements of increased self-efficacy are in the model, and self-efficacy may be an either an underlying or a primary outcome of play.

### SOCIAL COGNITIVE ELEMENTS IN BEHAVIOR CHANGE MEDIA

Bandura (2001) explains that SCT can be used in media in order to influence behavior change. This mechanism is what he calls a dual path of influence, in which the first path of influence is the direct influence of the message in the media on the population exposed to it. The second path of influence is on a person’s social circle, which in turn influences the person by creating an environmental facilitator and a social support.

The social cognitive elements, through SCT, have been validated through creation of media that results in positive behavior change outcomes. In a movement referred to as entertainment-education, 75 SCT-based media projects were internationally distributed in order to influence behavior change for HIV prevention, gender equality, family planning and adult literacy (Rogers et al., 1999). One of these projects, a radio show called Twende na Wakati, translated “Let’s Go with the Times” was created to increase the use of family planning practices. In a two year experiment, the show was aired in five regions of Tanzania, with one area running alternate material as a control. The show featured characters as models, with some characters modeling positive behavior and ending up with good lives (outcome expectations) while other characters modeled negative behaviors and suffered consequences. One negative character in particular had promiscuous sex and did not use condoms. His fate was to die of HIV. A positive character was a nurse who dispensed health information. The show also featured transitional characters who became more open to family planning practices as the show went on, embracing them by the end. Before the study ended, the show appeared to be influential. Several fans wrote letters to the show, saying that it was very educational and that they had spoken to their spouses about family planning because of it. The show increased self-efficacy in other ways as well, with one woman writing in and saying that the show influenced her and her husband to start a business. At the end of the study period, residents in experimental areas of Tanzania showed a marked increase in self-efficacy regarding family planning, and more residents had spoken to their spouses about using it than in the control area (Rogers et al., 1999).

SCT has also been used to successfully facilitate behavior change through video games. In a meta-analysis of 25 video game interventions to influence diet, exercise, and other health behavior, six games were specifically stated to be based on SCT, with three SCT-based games showing positive outcomes, and the other three not having completed the study at the time of the writing (Baranowski et al., 2008). In one notable game, called Packy and Marlon, players with juvenile diabetes decreased urgent care visits after playing the game (Brown et al., 1997). In the game, Packy and Marlon are elephants at a diabetes camp. They must go through several locations to find food and diabetes supplies that were scattered across the campground by enemy rats. Game locations include lakes, forests, mountains and even a haunted house. As Packy and Marlon reorganize their campground, they must use food and insulin logs to stay on their medication schedule and to eat according to their meal plans. Camp counselors ask them multiple-choice questions about diabetes self-care and how they would deal with several examples of social barriers. The treatment group gained self-efficacy regarding diabetes self-care, and decreased the number of urgent doctor visits related to their condition (Brown et al., 1997).

### MULTIPLE INTELLIGENCE ELEMENTS

The second set of elements in CBGD are based on Gardner (1983) theory of MI. MI is a multifaceted learning theory that integrates learning with activities designed to engage students according to their unique patterns of thought, called intelligences. According to Gardner, everyone has eight intelligences, and they form a profile describing how the person is intelligent. Unlike intelligence quotient (IQ) tests, there is no threshold under which a person is not considered intelligent. Each person has intelligence strengths and intelligence weaknesses (Gardner, 2003). This approach alone may increase self-efficacy because, in the MI framework, everyone is affirmed as intelligent, but in different ways.

### MULTIPLE INTELLIGENCES IN LEARNING

Like social cognitive elements, MI elements have been tested in real learning situations, with positive results. Gardner (1999) used the MI principles in a project called practical intelligence for school (PIFS). In the project, middle school students were taught using MI principles across various domains of learning. The project emphasized interdisciplinary problem-solving by investigating how various intelligences impacted a single issue. An example would be to determine whale swim patterns (spatial) by using the trajectory of swimming (math) and the sound of the whale calls (musical).

In the PIFS project, Gardner (1999) made several observations about effective learning using MI. The best learning happened when the information was presented in a real-world type of situation and there was a purpose for learning, and the information incorporated the student’s interests and allowed them to use their most salient abilities. When information was presented that was outside of the student’s abilities, then the students learned best when the environment was specific to the skill being learned and they were given time to focus on a single activity.

MI based teaching techniques have also been successful in classrooms around the world, transforming learning environments in areas as diverse as United States (Greenhawk, 1997), Turkey (Özdermir et al., 2006), and Malaysia (Sulaiman et al., 2011). When teachers were taught MI principles and asked to create MI based instruction, the lessons they created were activity-based and engaging for students, even though the content of the material remained the same as their traditional lessons. In one instance (Hopper and Hurry, 2000), teachers taught their students about their intelligences and helped them discover their strengths, then allowed them to use their intelligences to create their own homework assignments regarding a topic. One student who struggled with the traditional written homework chose to perform a dramatic video piece. Others created a board game. Students who had been formerly disengaged became active in the lessons.

In a formal study of MI performed in Turkey (Özdermir et al., 2006), two classes with fourth grade students were taught the same material by the same teacher. In the experimental class the teacher used MI based activities, while in the control class, only traditional teaching methods were used. Prior to the classes, students were tested on their intelligences (using the Teele Inventory of MIs) and on the subject to be taught (Diversity of Living Things).

In the traditional class, there was an emphasis on listening to the lecture, reading the blackboard, reading the textbook, and performing written work for homework. In the MI class, seven learning centers were created, representing each of the seven intelligences (naturalistic was not represented). During class time in the experimental group, each student spent 20 min in each of the seven learning centers, so that all students were exposed to all centers. Each learning center had its own activity relating to intelligence it represented (**Table 1**). Other than the MI vs. traditional activities, the content taught in each class was the same, and the learning centers were fitted with activities based on the subjects within the class, changing activities as the class moved through the curriculum. The classes spanned seven weeks, after which the students were re-tested on the subject exam. Two months post-class, they were tested on the subject again. Although the students had similar results on the Diversity of Living things pre-test, the MI group tested significantly better on both the immediate post-test and the 2-month retention test.

**Table 1:** MI centers in diversity of living things experimental class.

Learning center	Represented intelligence	Activity
**Personal work**	Intrapersonal	Work alone; examine a flower; draw petals
**Working together**	Interpersonal	Work in groups; discuss a flower; share and discuss drawings from Personal Work center
**Music center**	Musical	Compose and sing songs related to the parts of a flower; use instruments and create rhythmic chants
**Art center**	Visual/spatial	View and make charts and pictures of flowers; fashion parts of a flower out of play-doh
**Building center**	Body/kinesthetic	Act in dramatic stories involving flowers; use body language to explain flowers
**Reading center**	Verbal/linguistic	Read about the parts of a flower; write stories about flowers
**Math and science center**	Mathematical/logical	Play games and solve puzzles about flowers; perform problem-solving puzzles regarding flowers.

Educator and game designer Squire (2006) has suggested that educators need to design experiences rather than deliver content and that it benefits students to be in charge of their own design process. What Squire describes as an optimal learning environment is what teachers and students designed when instructed to use MI theory. If what Prensky (2001) describes as “tell-test” education is the problem and immersive, game-like environments are the answer, then MI theory is the bridge between the two.

### MULTIPLE INTELLIGENCE ELEMENTS IN CBGD

In CBGD, Gardner’s intelligences have been retranslated into game asset descriptions. So, while Gardner identifies musical intelligence, CBGD translates this as music and sound. Music is handled differently in a game than other sound effects. For instance, music is often used in a game introduction or as background music, while sound effects are used to help the player understand his or her environment. Sounds play to indicate that a player has done something correctly or incorrectly, or to indicate that something has changed and warrants investigation (like a sliding noise to indicate that an unseen passageway is now open). Another example is verbal intelligence, which has been translated in CBGD as words/language and narrative. Narrative refers to the underlying story of the game while words and language refer to other in-game elements. Words and language can be spoken in-game or shown on-screen. They are instantly understandable. Narrative, however, runs throughout the game and may require exposition, as more of the narrative is revealed as the player moves through the levels of the game.

While some of the MI elements are inherent to video games, like sound and graphics, they are included here as MIs in order to focus on their importance. While game designers may include these elements as part of entertainment, it is not clear that they understand that strategic inclusion of these elements may in fact facilitate learning. With this knowledge, designers can create elements that are designed to specifically contribute to the knowledge within the game. For instance, designers not using CBGD might include generic background music in a game for the purpose of establishing a general mood (i.e., happy; spooky) or simply to prevent the game from being silent. With CBGD, designers may think of how music can be used to enhance game knowledge, for instance having a score that gets slightly lower in volume the farther away the player ventures from a clue, and slightly louder the closer the player gets. Using music as a subtle in-game clue is a more active use of music to facilitate learning than generic background music. Music can also be used explicitly to communicate knowledge via song. With CBGD in mind, designers might choose to record songs with lyrics pertaining to the topic and include them either as explicit sing-a-longs or as subtle background music that holds clues and information the player needs.

The MI elements of CBGD are as follows:

#### Graphics

Graphics engage the visual/spatial intelligence. They are the pictures and graphic elements in a game. All graphics in a game have the potential to inform the player regarding the topic. Graphics include sets and locations as displayed in the game, as well as signs, objects, characters, etc. Graphics set the mood of the game and, while they don’t have to be in three dimensions or photo-realistically detailed, they do need to have a clear meaning. In CBGD, graphics can be especially important because international audiences may not understand words in the game, whether written or spoken.

#### Space/positioning

This is part of the visual/special intelligence. In CBGD, it refers to the way the player moves through the game environment. In some games, the player moves through a virtual environment, such as a dungeon, a room, or a maze. In other games, the player may move through a physical environment and must visualize his or her position relative to the position in-game. As more games are developed using body-sensors in lieu of traditional handheld controllers, the potential for using space and positioning in-games will increase.

#### Relationships/models

This refers to the use of interpersonal intelligence with in a game. Game relationships can refer to relationships with others surrounding the game, or relationships with characters within the game. This element is also present in the social cognitive sphere in the form of encouragement and social support.

While social support is mainly person-to-person, Bandura (2001) realized that media can act as a valid source of social support as well. Bandura realized that people often model themselves after others they know, but they also model people they don’t know if they are celebrities. Even fictional characters can act as behavior models.

Social support can take place in a massive multiplayer online game (MMO) guild online. MMO’s often contain several hundred players, working together as teams in order to complete game quests. Teams, called guilds in the MMO world, must compete with each other in order to win the game, which often involves being the first team to build or find an important relic. A serious game MMO could include offline meets as a part of the game, encouraging real-life activism. It could also take place by having a game that already includes a social aspect. For instance, Zombies Run () is a game that promotes fitness by sending alerts to the player’s phone that they are being chased by zombies. Groups can easily choose to meet at a certain place and be “chased” together.

Instead of creating a social network via the game, video games can garner social support by being associated with an existing social network. Many people take part in social networks because their friends are on them. Once on the network, some friends play games and invite their friends. Many social network games offer bonuses when a person invites a friend who joins, and some make getting new players integral to attaining game goals. Half the Sky: the game, is an example of a serious game that takes advantage of social media to garner donations and promote activism.

#### Music/sound

The musical element refers to any music in a game, whether background music, actual songs, or musical notes as part of a puzzle. Sound effects are also part of the musical element, as they can be used to communicate game information such as the correctness of an action, or off-screen events. Within MI theory, musical intelligence involves the ability to differentiate notes, pitch, and other elements of music. While people high in musical intelligence often play instruments, it is not necessary for the expression of musical intelligence. Simply recognizing elements of music is sufficient. For instance, a person with musical intelligence may be able to hear a song and distinguish the guitar, the violin, the piano, the bass, the harmonica and the harp as separate sounds in the music. Someone with a lesser musical intelligence may just hear a nice song, but not distinguish the instruments.

Music may also be a strong facilitator of learning as a construct. In 2011, an experiment was performed in which two fourth-grade math classes received the same math curriculum, but one group included songs related to the math concepts being taught. A standardized test was given at the beginning of the semester and again at the end. While both groups raised test scores by the end of the course, the experimental group raised scores by 57.7% while the control group raised scores by 47% (Yoho, 2011). While music is present in almost all games, it appears to function mostly as background ambience rather than being used to actively facilitate learning. Music as a facilitator of learning for serious games needs further exploration.

#### Narrative

The element of story in-games is one of Prensky’s (2001) six key elements of games. It is an expression of verbal intelligences within MIs theory, but it appears in CBGD as a separate element from words/language due to its unique influence in cognition and use in gaming. Narrative can be used several ways, one of which is the facilitator of situated cognition (Van Eck, 2006), in which the player is immersed into an environment or situation where the knowledge is presented in context. Using narrative with situated cognition allows a game to remain primarily a game, so that the player can focus on playing rather than on learning (Ciavarro, 2006). Although not designed for education, Nancy Drew video games () often include situated learning within a narrative. For instance, in one game, players must assemble DNA model to solve a puzzle. In a different game, periodic elements need to be matched with their symbols in order to solve another puzzle. Rather than an out-of-context quiz about educational content, players need to learn the content in order to solve the in-game puzzle.

#### Math/numbers

This is one component of the math/logic intelligence in MI theory. In CBGD, the math/numbers element refers to mathematical puzzles that require arithmetic, calculation, or geometry. While programming involves math, and therefore video games are built on a mathematical foundation, the math involved is not always made salient. In some games, it might be beneficial to “uncover” the math as part of the game. Also, including math puzzles in a non-math game can be a way of helping non-math oriented players become comfortable with math concepts.

#### Physical movement

This element represents the body/kinesthetic intelligence. While the space/positioning element refers to virtual spaces, the physical movement element refers to physical spaces. Games involving this element include dance, music band, and sports games that track the body rather than use a game controller. However, many of these games involve using the body for things the body would already do, such as dance or play a sport. The games add virtual companions and instruction along with a scoring system, which makes them video games. An interesting way of incorporating CBGD with physical movement is to use the game to have the body simulate a normally non-physical concept, such as forming letters or Roman numerals as a way of solving puzzles.

#### Logic/patterns

This is another component of the math/logic intelligence. In CBGD, this element refers to patterns involving reason rather than numbers. One of the most common formats for this element is the mystery game in which players must use clues, interviews, and environmental information to discover inconsistencies which lead to the capture of the culprit. However, other patterns are also common, such as seeing a picture of a landscape in a building and then recognizing that landscape later in the game in another form.

#### Words/language

This element is part of the verbal intelligence, and refers to the use of words and language within games. Language structure can be included in-games in the form of codes, words to unscramble, replace-a-word puzzles, or by having characters find letters or other literature in which words are missing. This element also refers to the use of on-screen text to communicate information. In some games, in-game encyclopedias and reference manuals are used to convey information. In others, books and scrolls can be found and opened in scenes where the information is needed. The words and language element also includes dialog, in which written text may or may not be accompanied by a voiceover.

#### Humor

Humor is not listed prominently in SCT or MIs theory, but it has a significant effect in both. When learning, humor can bring new perspectives to material, provide distance from the subject being learned, and create a sense of camaraderie with the instructor (Hovelynck and Peeters, 2003). Humor also enhances retention in learning situations. In a 14-week course, two classes were given identical video lessons with the exception that one set of lessons had integrated jokes that were directly related to the learning material. Both classes had the exact same final exams. Although the humorous elements were the only changes to the classes, the experimental class scored better on the tests. The experiment was replicated with the only change being that the first group included males and females, whereas the replicated groups were both female. The humor group once again scored higher than the control group (Ziv, 1988).

#### Nature

In MI theory, natural intelligence has to do with the ability to recognize patterns and elements in nature. This person is good at separating natural elements into categories, and picking out elements of nature from their surroundings. For instance, someone with natural intelligence may look at a rocky surface and recognize a fossil of an insect whereas someone less inclined toward this intelligence would just see rocks. In CBGD, this element can be enhanced with the use of actual photos of natural objects, and photo-realistic graphics and animations. More basic animations can also use the nature element by mimicking natural movements accurately even if the graphics look less than real.

#### Personal reflection

This represents the intrapersonal intelligence. A person with intrapersonal intelligence has a keen sense of self. He or she is aware of what is personally motivating and what is discouraging. This person is self-aware regarding abilities and limitations. In CBGD, this element can be expressed by using situations designed to provoke empathy. One example of this is the game Darfur is Dying (), in which the player character has to get water for his or her village while avoiding being captured by soldiers. Other examples are city simulation games, in which players must balance their budgets, the needs of the city and the desires of their citizens. Some simulation games even mimic conditions in real-life cities and nations, taking into account the unique needs of the citizenry along with budgetary and government constraints.

### THE ENJOYMENT PROCESS

In the quest to assist with the design of serious games, several authors have sought to explain what makes a game, what makes games enjoyable as well as educational (Gee, 2003; Squire, 2006; Prensky, 2001). Several aspects of those theories map directly to other elements of CBGD. For example, Prensky (2001) delineates six key elements of games that map almost exactly to: Rules, Goals and Objectives, Outcomes and Feedback, Opposition, Interaction, and Story (Prensky, 2001, p. 05–11). Rules can be an expression of Knowledge; Goals, Outcomes, and Opposition are mapped almost exactly; and Interaction can be a form of the relationships/role models element. In addition to finding elements that promote cognition, these researchers have found elements that contribute to enjoyment as well. Although several elements are included, there appears to be a few consistencies within elements listed as making a game enjoyable: engagement, challenge, flow, persistence, and mastery.

#### Engagement

Engagement is the attention-grabbing component of the enjoyment process. If the player is not engaged in the game, he or she will stop playing, thereby missing whatever learning or content is contained within it. While there are several ways of engaging players, some of the most salient include immersion (Gee, 2003), which involves making the player feel as if he or she has entered the game world. Immersion is often accomplished using realistic graphics, including details such as water that ripples and sound effects of birds and animals. Another way of engaging players is through the mechanism of first-person perspective, by which the player can identify with the character of the game as well as immersion in the game world, often even temporarily taking on the traits of the character they are playing (Christoph et al., 2009).

#### Challenge

Following engagement, players need a reason to continue playing the game. That reason is often challenge (Gee, 2003), which is consistently ranked as a primary motivation for play (Olson, 2010). Although life and work often present challenge, the other elements of CBGD combine within games to present challenge in a way that players find enjoyable. Another reason that challenge within games contributes to enjoyment is the presence of immediate and salient reward (Gee, 2003; Koster, 2003). The presence of challenge, feedback, and reward contribute so much to the enjoyment of games that they are being applied to other aspects of life, such as work; a process called gamification (Anderson and Rainie, 2012).

#### Flow

Many game theorists have noted the cyclical nature of challenge within games. Gee (2003) described a process in which players use prior information learned in the game to solve challenges they found later on, and recommended that easier challenges should come before more difficult ones. When skills and challenges are matched in a way that players are highly engaged and lose track of time, that is a state of flow (Csikszentmihalyi and LeFevre, 1989). Flow is not only a video game phenomenon, it is found while driving, interacting with family, and most of all through work (Csikszentmihalyi and LeFevre, 1989). Flow has also been found to be a result of self-efficacy and a facilitator of motivation (Csikszentmihalyi and LeFevre, 1989; Pavlas et al., 2010). Flow represents an optimal form of challenge, and, according to Koster (2003), it is what makes games fun.

#### Persistence

Persistence is one of the most important predictors of success in life (Duckworth et al., 2007), and games can make the process of building it enjoyable. In-game design, persistence is fostered by player mistakes and the game’s responses to them. Games are a way to practice making and recovering from mistakes because in-game consequences often have little to no bearing on real-life events. Furthermore, many games allow players to save before challenges are undertaken so that a failure takes them back to the point in the game where they can undergo the challenge again. Another aspect of games that encourages persistence is the long-term nature of gaming which allows a player to essentially freeze a challenge and walk away from it, returning later when he or she feels ready to try again.

#### Mastery

Mastery experiences are “win” experiences where the player is successful, and they are already an essential part of video games. Mastery is a component of self-efficacy, both as a contributor to self-efficacy and a result of it (Bandura and Schunk, 1981). In essence, people choose their challenges based on their feelings about their ability to accomplish them, and the consistent accomplishment of goals increases their perceived ability (Bandura and Schunk, 1981). Games provide a consistent mastery curve, which includes easy goals that are easily mastered and progressively leads to more difficult goals. Mastery of video games contributes to enjoyment on its own, but also may enhance enjoyment in a social aspect as players who master the more difficult levels of the game can gain respect from peers, or expand into helper roles for players on lower levels.

### CBGD AND MULTIPLE GAME PATHS

The elements of CGBD can be used to engage a wide variety of players by creating several game paths in a single game. For many games, there may be several puzzles, but only one type of puzzle, one solution, a single narrative and one “win” scenario. However, introducing choice can allow gamers to choose to play with elements that appeal to them, challenge them to strengthen weaker intelligences, or alternate between the two. For instance, allowing a choice of outcomes might appeal to gamers of varying self-efficacy, with one game path that allows for manual saving intermittently and a possibly more difficult challenge that only allows for auto-saves mid-level. Some games limit the number of retries before the challenge must be restarted.

For behavior change games, an effective strategy might be to allow players to choose their own barriers. This would allow them to focus on playing through the types of barriers they will most likely face in life. A player with a strong social network may choose environmental barriers that mimic their home or work environment or vice versa. This would increase situational cognition on an individual basis.

Apart from the goals themselves, CBGD would allow choices of game path. Although some game genres are non-linear by nature, as in adventure games, others, such as casual games, have repetitive levels. Even some adventure games have integrated puzzles. Usually these puzzles are presented in such a way that the player has only one and it must be completed before advancement. A choice of two or three obstacles, each based on a different intelligence element, would allow more players to master the game.

## EVALUATING CBGD

Cognitive behavioral game design combines social cognitive and MI elements, with an emphasis on narrative, and incorporating humor as appropriate. Understanding CBGD can help game designers and educators in their quest to create purposeful games. These principles can be incorporated into almost any type of game design in order to create a rich experience that appeals to many people, regardless of their own personal learning preferences.

CBGD can be evaluated via comparisons with other serious games that only employ a few CBGD elements with games which provide more. For instance, several educational games are merely digital quizzes, without a strong narrative. Others may operate like casual games in which players must stop and solve puzzles related to the primary social objective of the game, but the puzzles are not connected with the rest of the game play elements.

Another way is to compare a traditional serious game with a game that includes more CBGD elements via multiple paths. In both cases, experimental designs can reveal whether educational or social game goals were observed and retained by players of each type of game. Follow-up studies would indicate whether knowledge gained by playing CBGD designed games was retained for longer periods of time than knowledge gained by playing games without regard to CBGD. For activist games, CBGD games would hopefully predict real-life attempts at meeting social or behavioral game goals.

## GAME ANALYSIS THROUGH THE LENS OF CBGD: THE JOURNEYMAN PROJECT 3

In 1998, Presto Studios released the adventure game The Journeyman Project 3: Legacy of Time. CBGD is a new framework, so this game was not designed specifically with CBGD in mind, or even to be an educational game or to produce real-world reactions. However, upon analysis, this game features several components of CBGD. The design elements in the game are such that, if the content were changed to reflect real places and times, it could be quite a learning experience.

The game begins with video scenes that provide a brief back story. The main character is Gage Blackwood, who works with an intergalactic law enforcement agency. This is the character that the player represents throughout the game. The first puzzle of the game is a mini-game of sorts where the player must travel through 3-D landscapes and find a time code symbol in three different environments. The 3-D atmosphere and the time codes which are imprinted on objects are visually and spatially engaging. Each environment allows full panoramic motion so the player can see in all directions. Familiar landmarks allow the player to navigate similarly to being in a real environment. While searching for time codes, the player is shown the time code once and then given the choice to go to each environment. Maneuverability in each environment is limited, so the player will more than likely not get lost even if spatial intelligence is not his/her primary intelligence.

While most games include visual information, what sets The Journeyman Project 3 apart is the auditory in-game hint system. This hint system doesn’t reside in the help files where it needs to be accessed for specific hints, and it is not a library of information to read. The hint system in this game is an actual character named Arthur. Arthur is an artificial intelligence program that is built into the space suite that Blackwood wears. The game settings allow customization of Arthur so that he speaks a lot, making jokes about the environment as well as suggesting actions for Blackwood; speaks a little, leaving out the jokes and quips and sticking to hints only; or mute where he doesn’t speak at all unless the player clicks a hint bubble and requests information. If Arthur is muted, Arthur doesn’t speak until prompted. However, Arthur visually indicates when he has something to say, and what type of information he has available. If Arthur is simply making a comment, a speech bubble appears over his head. If Arthur has an idea or a suggestion for how to solve a puzzle, a light bulb appears. The player clicks the symbol for the information he or she wants to hear. Arthur physically resides in the bottom right corner of the screen, so the player can always access information without leaving the main screen. Not only does Arthur represent the word/language element, his presence includes social cognitive elements as well. He provides knowledge about the various environments, creates miniature goals and outcome expectations by providing ideas about how to solve puzzles, and provides a relational element by encouraging the player when successful actions are performed. Arthur’s helpful presence assists the player in completing game tasks, which also provides a sense of mastery, which can increase the player’s self-efficacy.

Another way that The Journeyman Project 3 is uniquely applicable to CBGD is the inclusion of relational and personal reflection elements. The player character is Gage Blackwood, but Blackwood is a time traveler who is wearing a space suit. Blackwood travels to Shangri La, Atlantis, and El Dorado. Each of these environments is in the ancient past, and the people there would not react well to seeing a man in a space suit. Therefore, in order to engage with anyone in each environment, Blackwood must capture the image of a native to the time and then allow his suit to shape-shift into the image of that person. Blackwood, disguised, can speak to anyone in that time period except the person he has shifted into. While Blackwood can only appear as one person at a time, every image he captures is stored into his inventory and he can change disguises at will. This creates an interesting relational aspect of the game. There are several times when a player needs to be a specific person in order to gain needed information. Part of the strategy of the game is to use relational elements to figure out which disguise is the most appropriate for each puzzle. For instance, in Atlantis, Blackwood discovers that there is a festival that the natives are preparing for, and a rebellion that the slave class is preparing for. The player is required to switch between noble characters that have access to the temple, and peasant characters who exchange information about the rebellion. Blackwood needs information and access to all areas in order to complete his mission. Of course, while Blackwood disguises himself as others, he has a mission of his own to keep in mind. Therefore, both the personal reflection element (Blackwood adhering to his own agenda) and the relational element (Blackwood choosing the right persona to get information) are necessary in order to successfully complete the game.

Other puzzles incorporate the other elements. The nature element is referenced in puzzles involving clay, olives and water. The space/positioning element is presented through tasks which involve physically maneuvering around in-game to get to strategic spots. One such puzzle involves repairing a bridge. Another involves sliding down a pole to get to a boat. Still another involves getting into a hot air balloon. There are also a few riddles, involving the logical element. The musical and math/numbers elements are combined into a single puzzle in which the player must click several musical scrolls in the correct order. The scrolls make different notes when clicked, but are also lined up in such a way that a person could decide to number them or label them in order to deduce the solution. Persistence is fostered when the player reaches an impasse and must go to another location in order to continue the game. There are three locations that can be accessed at will, so whenever a player gets stuck in one area, she can move to a different location and solve more puzzles. Items from all three locations are required to finish the game. The player can also save the game at any time in order to preserve game progress.

In essence, The Journeyman Project 3 has almost every element in CBGD, with the exception of physical movement. There is only mouse interaction in the game and no physical hands-on simulations. The technology for using the body as a game controller was not prevalent when the game was created or released. The game is not considered educational only because the three primary locations are fictitious. However, a game with the same storyline and game elements that used real historical events and real locations could prove to be incredibly educational. A modern premise with a call to action, such as a time traveler who has to warn a community about an upcoming food shortage, could induce players to investigate real-world solutions, such as urban farming.

## CONCLUSION

While there are several games that include serious topics, the inclusion of serious game elements is not enough to induce learning or real-world action. CBGD is the use of elements in SCT and MIs theory in an enjoyable way, for the purpose of creating games that induce real-world behavior change in the player. The social cognitive elements are knowledge, self-efficacy, goals/plans/outcome expectations, social support, and barriers. These elements can be incorporated into game design via the use of in-game libraries, NPCs, the moderation of difficulty for game levels, and even save and restore points.

Conventional education involves a form of passivity for students in that they are often expected to be quiet learners who simply absorb the content introduced by the teacher. However, the interactivity of game environments allow for the use of Howard Gardner’s expanded learning theory which involves MIs: logical/mathematical, musical, interpersonal, intrapersonal, visual/spatial, natural, body/kinesthetic, and linguistic. All of the intelligences can be used to enhance the elements in SCT. Game designers can create opportunities for players to gain knowledge, social support, and self-efficacy by incorporating the various intelligences into hints, puzzles, and game challenges.

## Conflict of Interest Statement

The authors declare that the research was conducted in the absence of any commercial or financial relationships that could be construed as a potential conflict of interest.
